# First molecular identification of the zoonotic parasite *Anisakis pegreffii *(Nematoda: Anisakidae) in a paraffin-embedded granuloma taken from a case of human intestinal anisakiasis in Italy

**DOI:** 10.1186/1471-2334-11-82

**Published:** 2011-03-31

**Authors:** Simonetta Mattiucci, Michela Paoletti, Francesco Borrini, Massimo Palumbo, Raffaele Macarone Palmieri, Vincenzo Gomes, Alessandra Casati, Giuseppe Nascetti

**Affiliations:** 1 Department of Public Health and Infectious Diseases, "Sapienza - University of Rome", P.le Aldo Moro, 5, 00185 Rome, Italy; 2 Department of Ecology and Sustainable Economic Development, "Tuscia University", Viale dell'Università s/n Viterbo Italy; 3 "Belcolle Hospital", Viterbo, Italy

## Abstract

**Background:**

Anisakiasis is an important fish-borne zoonosis provoked by larval stages of nematodes belonging to the genus *Anisakis*. The detection and identification of human infections is difficult. This is due to: a) the low specificity of the clinical features and symptomatology related to human infections; b) the paucity of diagnostic features of larvae found in granulomatous lesions characteristic of "invasive anisakiasis"; and c) the lack morphological characters diagnostic at the specific level when larvae of *Anisakis *are detected. Thus, molecular-based diagnostic approaches are warranted.

**Method:**

We have developed a PCR method that amplifies the DNA of *Anisakis *spp. in fixed paraffin-embedded tissues. This method was applied to a granuloma removed from a human case of intestinal anisakiasis in Italy. Specific primers of the mtDNA *cox2 *gene were used and sequence analysis was performed according to the procedures already established for species of *Anisakis*.

**Results:**

The sequence obtained (629 bp) was compared with those of the other species of *Anisakis *which have so far been genetically characterized and with sequences obtained from larval stages of *Anisakis *collected from the Mediterranean fish *Engraulis encrasicolus*. This enabled the genetic identification of the larva in the human tissue as *A. pegreffii*. This is the first instance of human intestinal anisakiasis diagnosed using PCR of DNA purified from a fixed eosinophilic granuloma embedded in paraffin.

**Conclusion:**

The case of human anisakiasis presented reinforces the pathological significance of the species *A. pegreffii *to humans. The molecular/genetic methodological approach based on mtDNA *cox2 *sequence analysis, described here, can allow easy and rapid identification of *Anisakis *spp. in formalin-fixed and paraffin embedded tissues removed from cases of either gastric or intestinal human anisakiasis.

## Background

Anisakiasis is a fish-borne zoonosis provoked by the larval ascaridoid nematodes of the genus *Anisakis *Dujardin, 1845. These nematodes have a complex life-cycle involving organisms at various levels of a trophic web in the marine ecosystem. Small crustaceans, mainly euphasiaceans, are the first intermediate hosts, whereas fish and squid are intermediate/paratenic hosts, and they reach the adult stage in the stomachs of marine mammals (mainly cetaceans) [1]. In fish, larvae are commonly localized on the surfaces of the visceral organs, body cavity, serous membranes and, occasionally, also in the muscle. Humans can accidentally be infected by the larvae when consuming raw, undercooked or improperly processed (e.g. marinated) parasitized fish and squid. In humans, these parasites do not mature, but they provoke the zoonosis termed anisakiasis. In such infections, these parasites can migrate from the gastrointestinal tract, become embedded in the gastrointestinal mucosa and submucosa, and initially cause discomfort and pain (gastric pain, vomiting) and, subsequently, an eosinophilic granuloma [2]. Based on the location of the granulomatous lesions, various types of human anisakiasis have been identified, most being either gastric or intestinal. In these situations, larvae penetrate the tissues of the alimentary tract and cause severe pathology, even at the extra-gastrointestinal level [2,3]. Oropharyngeal cases of human anisakiasis have been also reported [2]. From the histopathological point of view, anisakiasis may be classified into four stages: i) phlegmon formation; ii) abscess formation; iii) abscess-granuloma formation; and iv) granuloma formation [2]. In the recent years, it has been suggested the occurrence of allergy symptoms associated with *Anisakis *infections. Indeed, hypersensitivity cases have been particularly noted so far in Spain, and the occurrence of a gastro-allergic anisakiasis has been documented [4-6].

This zoonosis, after first being reported in the Netherlands [7], has been noted particularly in Japan, but also in European countries where its occurrence has been related to an increase in the popularity of raw and/or undercooked fish. In Italy, in recent years, several cases have been reported [8-17]; most of these were based on histopathological findings, but in three cases only molecular diagnoses have been made on larval nematode extracted by gastroduodenoscopy [10,16,17].

For any parasitological survey and epidemiological study to be carried out on these worms, the exact identification (to the specific level) of the species occurring in a geographical area represents the first parameter to be considered. Genetic-molecular methods are particularly useful in this regard, since larval stages of *Anisakis *are not recognizable by morphological characters. The systematics of the species of *Anisakis *has been clarified in recent decades by the application of such molecular methodologies [1,18-34]. The actual taxonomy of the species so far recognised in this genus has been reviewed recently [1,24].

Among the nine species of *Anisakis *so far characterized genetically, the two sibling species, *A. simplex *(*s.s*.) and *A. pegreffii *have been identified as agents of human anisakiasis [10,16,17,30].

Molecular methods have so far been applied only to nematode larvae obtained by endoscopy. Furthermore, only morphology has been used to try and recognize larval nematodes in histological sections of surgically removed granulomas formed in gastric and/or intestinal tissues. Such identification has not always been possible due to the poor conservation of worms present in histological sections. Even when attempted, identification has been possible only to the generic level. Therefore, this paper aims to: *i) *provide a method based on PCR-DNA and the sequencing of the mtDNA *cox2 *gene for the identification of larval stages of *Anisakis *from paraffin-embedded tissues after the surgical removal of the granuloma formed by the larva; and *ii) *identify for the first time, to the species level, an *Anisakis *larva recovered from a case of human intestinal anisakiasis.

## Case presentation

A man was admitted to the Emergency Department of the "Belcolle Hospital" in Viterbo, Italy, complaining of acute abdominal pain in the lower right quadrant, nausea and emesis lasting several hours. He was admitted with suspected appendicitis, and physical, laboratory and radiological findings also suggested acute appendicitis. During the surgical inspection, a lesion of the caecum was detected. The caecum was dilated and exhibited an edematous and inflamed mucosa. Therefore, a right colectomy was performed. The surgical specimen consisted of a tract of the small bowel; the ileo-caecal valve was stenotic and the mucosa appeared to be extensively edematous with severe thickening of the wall of the caecum. Human tissue sample from the organ showing the gross lesion was fixed in 10% neutral buffered formalin, embedded in paraffin, sectioned at 6 μm and stained with haematoxylin and eosin. Sections revealed in the submucosa, a granuloma surrounding the larva of a nematode. Some of the obtained sections were analyzed using light microscopy and with the aid of a drawing tube.

### DNA extraction from tissue paraffin-embedded

One of the histological sections obtained from the paraffin-embedded tissue was used for the DNA extraction. This was carried out applying a DNA extraction technique from paraffin-embedded tissue [35], with the following modified procedures. The removal of paraffin was carried out by adding 1 ml of xylene for 30 min to the microtube containing the tissue section, followed by two washing steps (30 min each) using 100% and 75% ethanol. The tissue mixture was then washed with PBS for 15 min, which was repeated three times. The lysis buffer (Proteinase K 2 mg/ml, 50 μl, 1 M Tris-HCl solution, 10 μl; 0.5 M EDTA, 2 μl; 10% SDS, 100 μl; distilled water, 838 μl) was added and the mixture incubated at 52°C overnight. 500 μl of phenol, chloroform and isopropanol alcohol, in the proportion 25:24:1, respectively, were then added to the de-waxed tissue. The sample was mixed by vortex and centrifuged for 10 min at 13.000 rpm, and 1 ml of chloroform was added to the supernatant, transferred to an autoclaved microtube and centrifuged for 5 min at 13.000 rpm. Subsequently, 0.1 ml of 3 M sodium acetate was added, followed by 1 volume of isopropanol, and the mixture incubated at -20°C overnight. The precipitated DNA was then centrifuged at 12.000 rpm and then washed with 75% ethanol. The white pellet was dried at room temperature overnight for 10-15 min. The DNA pellet was suspended in 50 μl of TE buffer (pH = 8) and then stored at -20°C.

### PCR condition and DNA sequencing

Identification to the species level was carried out using a 629 bp fragment of the mitochondrial Cytochrome Oxidase 2 (*cox2*) gene. The *cox2 *gene from *Anisakis *spp. was amplified using the primers 211F 5'-TTT TCT AGT TAT ATA GAT TGR TTY AT-3' and 210R 5'- CAC CAA CTC TTA AAA TTA TC-3' from Nadler & Hudspeth [36] spanning the mtDNA nucleotide position 10,639-11,248, as defined in *Ascaris suum *[GenBank X54253]. PCR (polymerase chain reaction) amplification was carried out in a volume of 50 ml containing 30 pmol of each primer, MgCl_2 _2.5 mM (Amersham Pharmacia Biotech. Inc., Piscataway, NJ), PCR buffer 1 X (Amersham Pharmacia Biotech. Inc., Piscataway, NJ), DMSO 0.08 mM, dNTPs 0.4 mM (Sigma-Aldrich, St. Louis, MO), 5 U of *Taq *Polymerase (Amersham Pharmacia Biotech. Inc., Piscataway, NJ) and 10 ng of total DNA. The mixture was denatured at 94°C for 3 min, followed by 34 cycles at 94°C for 30 sec, 46°C for 1 min and 72°C for 1.5 min, followed by post-amplification at 72°C for 10 min. The PCR product was purified using PEG precipitation, and automated DNA sequencing was performed by Macrogen Inc. (Seoul, Korea) using primers 210R and 211F. The sequences obtained were compared with those already obtained for all of the *Anisakis *spp. reported above and previously deposited by us in GenBank with their accession numbers. They are the following: *A. simplex *(*s.s*.) [DQ116426], *A. pegreffii *[DQ116428], *A. simplex *C [DQ116429], *A. typica *[DQ116427], *A. ziphidarum *[DQ116430], *A. physeteris *[DQ116432], *A. brevispiculata *[DQ116433], *A. paggiae *[DQ116434] and *A. nascettii *[DQ116431], as reported in our previous papers [24]. The *cox2 *sequences were aligned using Clustal X [37]. Phylogenetic analysis was performed by MEGA 4.0 [38], using Maximum Parsimony (MP) and Neighbour-Joining (NJ) based on *p-distance *values. The reliabilities of the phylogenetic relationships were evaluated using nonparametric bootstrap analysis [39] for the MP and NJ trees. Bootstrap values ≥70 were considered well supported [40]. Additionally, the DNA obtained from the human tissue extraction was also compared with other *A. pegreffii *specimens collected from a fish, the anchovy *Engraulis encrasicolus *from the Mediterranean Sea [24,41]. A sequence at the mtDNA *cox2 *of *Pseudoterranova decipiens *(*s.s.*) from *Phoca vitulina *was included as the outgroup to root the *Anisakis *phylogenetic tree.

## Results

**Figure 1:**
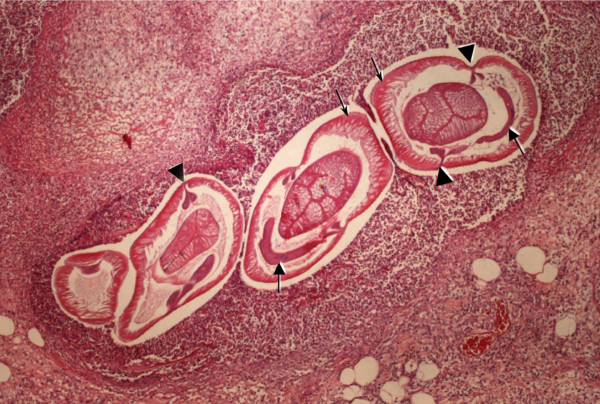
**Transverse sections of the *Anisakis *larva in the submucosa of the intestinal wall**. Polymyarian muscle cells (thin arrows), separated into four quadrants by the chords, showing two wing-like distal lobes (arrow heads). Excretory cells are banana shaped (thick arrows) and situated ventrally to the intestine (H&E staining; original magnification ×100).

The histological examination revealed a transmural inflammation with marked infiltration of eosinophils, histiocytes, lymphocytes and plasma cells across all layers of the bowel wall and in the peri-intestinal fat tissue. In the submucosa, where the infiltration of eosinophils was most evident, a parasitic nematode in a good state of preservation was found. The nematode had a diameter of 0.50 × 0.30 mm, with a thin (~10 μm) cuticle lacking lateral alae. Polymyarian muscle cells, separated into four quadrants by the chords were visible, with two wing-like distal lobes (Figure 1). The intestine was circular, with a triangular lumen and 60-70 tall columnar epithelial cells disposed radially. Excretory cells (renette cells) were banana-shaped and situated ventrally to the intestine. There was no ventricular appendix and/or intestinal caecum present (Figure 1). The nematode was identified as a larva belonging to the genus *Anisakis*.

**Figure 2:**
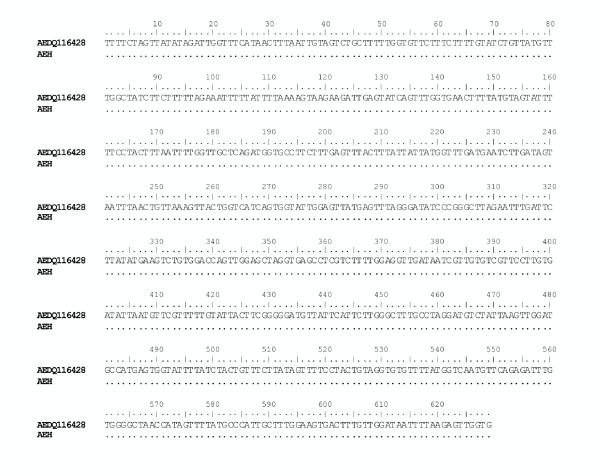
**Alignment of the mtDNA *cox2 *(629 bp) sequences of *Anisakis *spp., by using BioEdit **[42]. mtDNA *cox2 *(629 bp) sequence of the *Anisakis pegreffii *larva identified from the paraffin-embedded tissue of human intestinal anisakiasis (code: AEH), with respect to the other *Anisakis *spp. which have previously been sequenced [24] and deposited in GenBank. Accession numbers are reported in the text. Dots indicate identity.

**Figure 3:**
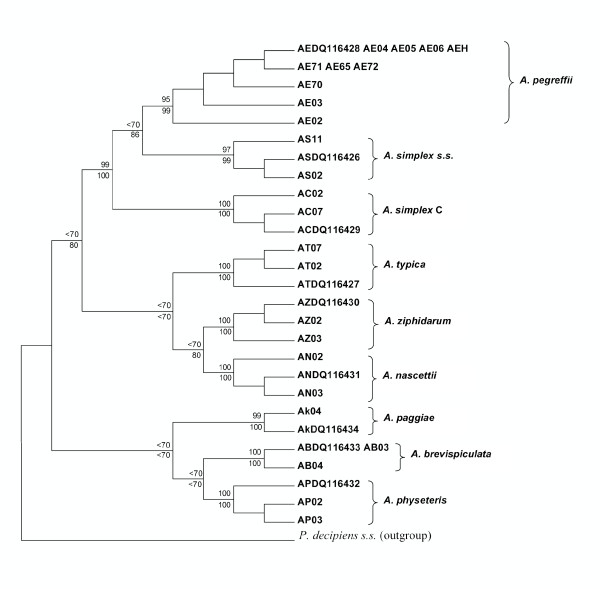
***Cox2 *derived Maximum Parsimony (MP) and Neighbour-Joining (NJ) condensed consensus trees inferred by MEGA4.0 for the specimen of *Anisakis pegreffii *larva from the human intestinal case (code: AEH) sequenced for the mtDNA *cox2 *(629 bp)**. The MP tree was obtained by the bootstrap method with a heuristic search. There were 213 polymorphic sites and 178 parsimony-informative sites. Sequences of *A. pegreffii *from *Engraulis encrasicolus *are reported with the codes: AE71, AE65, AE72, AE70. Sequences of *A. pegreffii *from our previous studies are reported with the codes AE03 and AE02 [28]. Number of bootstrap replicates = 1000; bootstrap percentages of the clades ≥ 70 are shown at the nodes, with MP and NJ values are reported above and below the nodes, respectively. Sequences from the other *Anisakis *spp. are those previously analyzed by us [24,28] and deposited in Genbank. Accession numbers are reported in the text. *Pseudoterranova decipiens *(*s.s.*) was used as the outgroup.

The fragment of *Anisakis *larva obtained from a tissue sample, fixed in formalin, embedded in paraffin, was perfectly sequenced at the mtDNA *cox2 *gene: a sequence of 629 pb was obtained. The specimen of *Anisakis *sp. collected from the human intestinal granuloma matched perfectly the previously studied sequences of *A. pegreffii *[28,24,41]; it was deposited in GenBank under the accession number DQ116428 (Figure 2). Furthermore, the *A. pegreffii *specimen of human origin clustered in the same well-defined and well-supported clade in both the Maximum Parsimony (MP) and Neighbour-Joining (NJ) analyses (Figure 3) with those specimens of the species *A. pegreffii *which had previously been sequenced [24,28,41] and deposited in GenBank. In addition, the larval *A. pegreffii *from the human case clustered, using MP and NJ analyses (Figure 3), in the same well-supported clade with the *A. pegreffii *larvae collected from anchovies (*Engraulis encrasicolus*) in the Mediterranean Sea. A very low value of genetic differentiation at the mtDNA *cox2 *level (*p distance *= 0.001) was found between the sample of *A. pegreffii *from the human infection and those samples of *A pegreffii *so far sequenced from the Mediterranean Sea. Nevertheless, this result has allowed the identification of *A. pegreffii *as a zoonotic agent of a human intestinal case of anisakiasis within an eosinophilic granuloma.

## Discussion

The paucity of documented cases of anisakiasis in Italy contrasts markedly with the presence of these parasites in fish species [1] which are increasingly consumed in Italy as raw or marinated sea-food. Clearly, this mismatch suggests that human infections with *Anisakis *in Italy could be significantly underestimated.

After human ingestion, larvae could be either expelled in vomit or stools, or they could penetrate the wall of the gastrointestinal tract. As a consequence of the latter, a local granulomatous reaction surrounds the invading larva [2,3]. Such chronic intestinal anisakiasis can develop into abdominal peritonitis or intestinal occlusion and, hence, become a surgical problem - as in this report. Subsequent histological examination in the present case revealed the presence of an *Anisakis *larva.

In previous cases of gastric [8], intestinal [14,15], extra-intestinal [9] and gastrointestinal [11,12] human anisakiasis in Italy, the histopathological findings disclosed an *Anisakis *larva, but no identification to species level was possible. So far in Italy, only three human cases have been identified by molecular methods; in these cases, this was enabled by the removal of *Anisakis *larvae from the stomach by endoscopy followed by PCR-RFLP analyses of the ITS of rDNA or sequences analysis of the mtDNA cox2 gene [10,16,17]. Significantly, in these cases fresh fragments of larvae were available for analysis.

In contrast we report, for the first time, the identification of an *Anisakis *larva sequenced from histological tissue previously fixed in formalin and subsequently embedded in paraffin. This represents a further report of *A. pegreffii *as a zoonotic agent of human anisakiasis in Italy, and the first case of its identification to the species level in an intestinal anisakiasis in Italy. One previous human case referring to *A. pegreffii *was reported in Japan [30], among several cases in the same geographical region where *A. simplex *(*s.s.*) was determined to be responsible [30]. The identification of *A. pegreffii *as a causative agent of this case of intestinal anisakiasis in Italy is in accordance with the widespread occurrence of the parasite species, at a larval stage, in various fish and squid species in Italian seas [1].

The medical history of the patient in the present case indicated the consumption of raw, marinated anchovies approximatively two months before the onset of abdominal pain. This fish species is frequently infected with *A. pegreffii *in the Mediterranean Sea, with an average prevalence of infection in the Tyrrhenian Sea P 1.00% and an average mean intensity, Im 1.00 (our unpublished data). In this fish species, the relative proportion of *Anisakis *larvae occurring in the flesh, i.e. in its edible parts, was 0.01% of the total number of larvae recovered in the infected fish [our unpublished data]. Since in this human case and in other reports, the medical histories of the patients indicated prior consumption of marinated anchovies, we infer that this sea-food preparation could be an important source of infection of anisakiasis in Italy.

## Conclusion

The molecular/genetic methodological approach described here easily and rapidly identifies *Anisakis *spp. in formalin-fixed and paraffin embedded tissues removed from cases of either gastric or intestinal human anisakiasis. This will facilitate a better estimation of the epidemiological role of the zoonotic species *A. pegreffii *in the Italian population and will also allow analysis of previously removed granulomas currently residing in histology archives.

The case of human anisakiasis presented reinforces the pathological significance of the species *A. pegreffii *to humans. Furthermore, the molecular characterization, based on the mtDNA *cox2 *gene, will enable investigations into the possible occurrence of different haplotypes of those species of *Anisakis *associated with the differential pathological aspects of this fish-borne zoonosis (anisakiasis) in humans. In this respect, the high genetic polymorphism (haplotypes) so far observed in the mitochondrial DNA *cox2 *gene in *Anisakis *spp. [24] reveals that its sequence analysis offers not only valuable and efficient molecular tools for the recognition of sibling species of zoonotic *Anisakis *larvae as independent genetic lineages [24,28], but also provides, for future analyses, additional genetic characters useful for molecular epidemiological and pathological approaches to the study of human anisakiasis [6].

## Competing interests

The authors declare that they have no competing interests.

## Authors' contributions

SM, MP and GN performed the molecular identification of the parasite, analyzed the genetic data, reviewed the literature and wrote the MS. FB performed the histological examination; RMP, MP, VG and AC managed the patient. All authors have read and approved the content of the manuscript.

## Pre-publication history

The pre-publication history for this paper can be accessed here:

http://www.biomedcentral.com/1471-2334/11/82/prepub
